# Photoinduced damage of AsLOV2 domain is accompanied by increased singlet oxygen production due to flavin dissociation

**DOI:** 10.1038/s41598-020-60861-2

**Published:** 2020-03-05

**Authors:** Martina Petrenčáková, František Filandr, Andrej Hovan, Ghazaleh Yassaghi, Petr Man, Tibor Kožár, Marc-Simon Schwer, Daniel Jancura, Andreas Plückthun, Petr Novák, Pavol Miškovský, Gregor Bánó, Erik Sedlák

**Affiliations:** 1 Department of Biophysics, Faculty of Science, P.J. Šafárik University, Jesenná 5, 041 54, Košice, Slovakia; 2 BioCeV - Institute of Microbiology, Průmyslová 595, 252 50, Vestec, Czech Republic; 3 Center for Interdisciplinary Biosciences, Technology and Innovation Park, P.J. Šafárik University, Jesenná 5, 041 54, Košice, Slovakia; 4 Department of Biochemistry, University of Zürich, Winterthurerstrasse 190, CH-, 8057 Zurich, Switzerland; 5 SAFTRA photonics Ltd, Jesenná 5, 041 54, Košice, Slovakia

**Keywords:** Biochemistry, Biophysics

## Abstract

Flavin mononucleotide (FMN) belongs to the group of very efficient endogenous photosensitizers producing singlet oxygen, ^1^O_2_, but with limited ability to be targeted. On the other hand, in genetically-encoded photosensitizers, which can be targeted by means of various tags, the efficiency of FMN to produce ^1^O_2_ is significantly diminished due to its interactions with surrounding amino acid residues. Recently, an increase of ^1^O_2_ production yield by FMN buried in a protein matrix was achieved by a decrease of quenching of the cofactor excited states by weakening of the protein-FMN interactions while still forming a complex. Here, we suggest an alternative approach which relies on the blue light irradiation-induced dissociation of FMN to solvent. This dissociation unlocks the full capacity of FMN as ^1^O_2_ producer. Our suggestion is based on the study of an irradiation effect on two variants of the LOV2 domain from *Avena sativa*; wild type, AsLOV2 wt, and the variant with a replaced cysteine residue, AsLOV2 C450A. We detected irradiation-induced conformational changes as well as oxidation of several amino acids in both AsLOV2 variants. Detailed analysis of these observations indicates that irradiation-induced increase in ^1^O_2_ production is caused by a release of FMN from the protein. Moreover, an increased FMN dissociation from AsLOV2 wt in comparison with AsLOV2 C450A points to a role of C450 oxidation in repelling the cofactor from the protein.

## Introduction

Flavin mononucleotide (FMN) belongs to a group of efficient endogenous photosensitizers in cells with rather high singlet oxygen, ^1^O_2_, quantum yield (Φ_Δ_) within the range 0.51–0.65^1,2^. Depending on FMN concentrations and concentrations of available oxygen, the flavin(s) can be even more effective ^1^O_2_ generators than exogenous porphyrins used for cell killing in photodynamic therapy (PDT). To minimize the potential deleterious effect of flavins to cells, the isoalloxazine moiety of flavin cofactors is typically deeply buried in the protein core of flavoenzymes^3^ or storage proteins^4^.

Singlet oxygen, the lowest energy excited electronic state of molecular oxygen, belongs to the group of reactive oxygen species (ROS), which includes superoxide anion (O_2_^•−^), hydrogen peroxide (H_2_O_2_), and hydroxyl radical (HO^•^), enabling to oxidize and/or oxygenate many biologically relevant molecules^5,6^. Singlet oxygen can be produced in a variety of ways by physical mechanisms, including energy transfer from the excited triplet states of particular chromophores to molecular oxygen^7^, or by chemical mechanisms as one of the products of peroxidase enzymes^8^. In biological systems, ^1^O_2_ is usually generated by electronic energy transfer from an excited state of a photosensitive molecule, so-called photosensitizer (PS), to ground state O_2_^6^. The high reactivity of singlet oxygen towards biological molecules is relevant in the context of PDT^9^ and chromophore-assisted laser inactivation (CALI) of proteins and cells^10,11^.

Studies addressing the behavior and action of ^1^O_2_ have been performed for decades, but there is still a limited knowledge on the spatially- and temporally-dependent ^1^O_2_-induced cell signaling processes^12,13^. An encapsulation of a photosensitizer in a protein matrix, thus forming a genetically-encoded photosensitizer, facilitates ROS production with (i) molecular level spatial control via protein targeting, and (ii) temporal and dose control by the incident light^2,13,14^.

On the other hand, the improved control regarding ^1^O_2_ production by using genetically-encoded photosensitizers leads to attempts to utilize them as antimicrobial agents^15,16^ or in PDT^17^. Up to now, more than 400 individual compounds have been recognized as possible candidates for use as PSs^9^. However, a large fraction of the small organic PSs has unsuitable physico-chemical properties such as low solubility and stability in aqueous solvents, and inherent low specificity for targeted diseased tissue. Consequently, such compounds exhibit a certain toxicity to other, healthy tissues^18,19^. Alternatively, the use of proteins as genetically encoded ^1^O_2_ generators offers new ways of designing, synthesizing, and targeting of biomacromolecules containing PS^20^.

Recently, significant efforts have been invested into the design of protein PSs containing FMN due to its high value of Φ_Δ_^15,21–23^. On the other hand, genetically encoded fluorescent tags have inherently very low efficiency of ^1^O_2_ production (Φ_Δ_ < 0.09)^15,20,24,25^. These observations point to a strong effect of the surrounding protein matrix on ^1^O_2_ production efficiency by the chromophore^21^.

Two major ways how the protein environment diminishes the yield of ^1^O_2_ production have been identified: (i) inefficient diffusion of molecular oxygen through the protein scaffold to the site of PS localization and (ii) quenching of the excited state of PS, e.g. FMN triplet state, by the protein environment^23^. In fact, efficiency of the ^1^O_2_ production in miniSOG (mini-singlet oxygen generator; Φ_Δ_ = 0.03–0.05)^21,26^, engineered from the FMN-containing LOV2 domain of *Arabidopsis thaliana* phototropin 2^27^, upon chemical denaturation increased over 10-fold in comparison with its native form^21^. Consequently, these observations led to efforts to develop protein PSs with improved ^1^O_2_ production, such as SOPP (singlet oxygen photosensitizing protein; Φ_Δ_ = 0.19–0.26)^26^ and particularly SOPP3 with Φ_Δ_ = 0.60, comparable to that of free FMN^23^. These improved variants of miniSOG were obtained by identification and replacement of amino acids responsible for: (i) steric barriers for oxygen diffusion toward the PS, (ii) quenching of FMN triplet state by electron transfer, and (iii) quenching of produced ^1^O_2_ by chemical reactions^23,26^.

In line with these observations is a finding of ~10-fold increase of the Φ_Δ_ value in miniSOG after its irradiation^23^. This finding was explained as the result of progressive photoinactivation of certain amino acids, such as His, Met, Phe, Trp, and Tyr, that are responsible for ^1^O_2_ quenching^5,8,28^ and/or the buildup of FMN photoproducts^21^. Indeed, very recently Torra *et al*.^29^ showed that the irradiation of miniSOG leads not only to oxidation of several residues, which are possible electron donors to FMN, but also to phototransformation of FMN to lumichrome, which results in facilitation of the access of molecular oxygen to the isoalloxazine ring of FMN.

In this work, we present detailed analysis of cumulative irradiation of two forms of LOV2 domain of phototropin 1 from *Avena sativa* (AsLOV2), wild type (wt) and its variant with replaced cysteine 450 (the numbering correspond to the original sequence of the LOV domain in phototropin 1) for alanine (C450A). These two variants differ by an ability of light-induced conformational change, which is induced by covalent bond forming between the thiol group of C450 and C(4a) on the isoalloxazine ring. While AsLOV2 is able of photoswitching, the variant C450A, due to removing of the thiol, has lost this property. The AsLOV2 primary structure is more than 80% identical with miniSOG. Our results clearly show different kinetics of FMN triplet states of the AsLOV2 and its variant, increased efficiency of ^1^O_2_ production as a function of irradiation time as well as oxidative modification of both proteins. Based on these observations, we conclude that the irradiation-induced increase of ^1^O_2_ production in the AsLOV2 variants is due to a release of FMN to solvent as a result of oxidative modification of certain amino acids in the AsLOV2 structure. Our results suggest a new approach towards designing an efficient protein photosensitizer as a carrier of a chromophore that can be subsequently released by irradiation of the protein at the site of its action.

## Experimental methods

### Cloning, expression and purification of the AsLOV2 domain

Wild type AsLOV2 as well as variant AsLOV2-C450A were expressed in *E. coli* strain BL21(DE3). The bacterial cells were grown at 37 °C in ampicillin containing (100 µg/ml) TB medium until they reached OD_600_ ~0.6–0.8. Protein expression was induced by adding isopropyl β-D-galactopyranoside (100 µM final concentration) following a temperature downshift from 37 °C to 25 °C. Expression was carried out in darkness overnight at 25 °C. The proteins were purified using metal ion affinity chromatography (Ni-NTA Superflow, Qiagen). The sequence of the final construct of AsLOV2 contains an N-terminal His_10_-tag, followed by amino acids 404–547 of AsLOV2 according the original numbering of phototropin 1. After IMAC purification they were run on a Superdex 75 Increase, 10/300 GL, size exclusion column and concentrated in 20 mM TrisHCl buffer, pH 7.8. All steps were performed in darkness. The ratio of absorbance at 280 nm/477 nm of the final protein was ~2.5, suggesting the absence of AsLOV2 apoform^30^.

### Sample irradiation (by laser) and singlet oxygen phosphorescence

Samples (1.2 ml) containing 25 μM protein were placed in a 10 × 10 × 40 mm quartz cuvette equipped with an overhead-type glass stirrer and kept at ~30 °C. A laser system consisting of a pulsed optical parametric oscillator (OPO) (GWU basiScan-M) pumped with the third harmonic of a Nd:YAG laser (Spectra-Physics, Quanta-Ray, INDI-HG-10S) was used to excite the samples. The OPO wavelength was tuned to 475 nm matching the absorption maximum of the AsLOV2 protein. The repetition rate of the 5–7 ns long laser pulses was set to 10 Hz. The 3 mm diameter laser beam was focused to the cuvette by means of a 200 mm lens. The average laser power on the sample was 0.9 mW. The phosphorescence signal of singlet oxygen passed through a 1250–1300 nm band-pass filter and was detected with a photomultiplier tube (Hamamatsu H10330A-75) operated in photon counting mode. A multichannel scaler PCI card (Becker & Hickl, MSA-300) was used to acquire the phosphorescence time course. In order to suppress the background signal originating from the optical components, the emission signal was measured with two additional band-pass filters, in the 1200–1250 nm and the 1300–1350 nm spectral regions. The background signal was assumed to have a slowly varying wavelength dependence in the covered spectral range. The resulting singlet oxygen phosphorescence was calculated by subtracting the average of the two auxiliary measurements (acquired in the adjacent spectral regions) from the signal measured in the 1250–1300 nm spectral range. The background was efficiently suppressed this way. Throughout the experiments an average of 2500 laser pulses was detected with each filter consecutively. The time needed for a single measurement set (three band-pass filters) was 12.5 min. The total irradiation time was 75 min.

### Measurements of FMN triplet state lifetime

An additional 633 nm cw laser was added to the optical setup to monitor the FMN triplet state lifetime in a flash-photolysis experiment^31^. The polarization of the cw laser was oriented at the magic angle with respect to the excitation beam polarization. The laser beam was passing through the sample area excited with the pulsed laser. The time-resolved absorption at 633 nm was measured with an avalanche photodiode (Thorlabs, APD110A2) connected to a digitizing oscilloscope (Tektronix, DPO 7254). The average signal of 2500 laser pulses was acquired consecutively throughout the irradiation experiment. Eighteen decay curves were measured during the 75 minutes interval. The protein concentration was 25 µM.

### Determination of a light-induced released of FMN

Relative amounts of released FMN from AsLOV2 wt and AsLOV C450A were determined by FMN fluorescence after filtration using 10 kDa cut-off filter tubes. Each sample, i.e. non-illuminated and illuminated AsLOV2 wt and AsLOV2 C450A, 900 µl of 10 µM protein, was loaded into Amicon Ultra Centrifugal filter tube and centrifuged for 5 min at 7500 *g*. After the spin, the collected flow-through of each sample was measured for FMN fluorescence.

### Spectral analysis

Different spectroscopic techniques have been used to follow the structural changes of AsLOV2 wild type and variant C450A after irradiation with blue light. All spectra were recorded at room temperature.

*Ultraviolet and visible absorption spectra* were obtained with a UV-2401PC UV-Vis spectrophotometer (Shimadzu). Protein concentrations were calculated by using an extinction coefficient of ε_447_ = 13800 M^−1^·cm^−1^ for oxidized FMN^32^. The measurements were performed in quartz cuvettes with 1 cm pathlength. The protein concentration was 25 µM.

*Fluorescence emission spectra* were recorded with a RF-5301PC spectrofluorophoto-meter (Shimadzu). The emission spectra of FMN and tryptophan were measured by using excitation wavelengths at 445 nm and 295 nm, respectively. For obtaining fluorescence spectra, a protein concentration of 10 µM was used.

*Circular dichroism spectra* measurements were performed by Jasco 810 (Jasco). The protein concentration used in CD measurements was 10 µM. The measurements in the far-UV and the near-UV spectral regions were performed in quartz cuvette with 1 mm and 1 cm pathlengths, respectively.

### Adduct decay kinetics measurements

For light-induced adduct formation accompanied by absorbance changes in the spectral region 425–520 nm, we used a photographic flash (Canon) as a light source. Adduct decay kinetics were measured by following absorbance at 447 nm in quartz cuvettes with 1 cm pathlength. A UV-2401PC UV-Vis recording spectrophotometer (Shimadzu) was used. In all measurements, the protein concentration was 25 µM.

### Molecular modeling

The Maestro/Desmond^33,34^ combination of programs has been used for model building, energy minimization, MD simulations and analysis of simulation results. The OPLS-2005 force field (the up-to-date version of the OPLS force field family^35–42^) was used to carry out the simulation studies. The two AsLOV2 protein structures (PDB ID: 2v0u and 2v0w) were downloaded from the Protein Data Bank, updated in Maestro using the “Protein Preparation Wizard”. The proteins were solvated (SPC water model^43^; the water molecules were added within 1 nm buffer around the protein, creating thus a 6 × 6 × 7 nm sized solvent box) and the resulting structures were then minimized. The final structures were equilibrated and submitted for 5 ns NPT (pressure at 1.01325 bar) simulations with the Desmond program at 300 K. Molecular geometries resulting from simulations were saved at 0.5 ps intervals and were visualized within the Maestro and VMD^44^ programs and were used for further analysis. The cystine thiol of C450 of AsLOV wt (PDB ID: 2v0u) was oxidized to SOO^−^ and the resulting structures were also solvated, and NPT simulated for 5 ns in Desmond as described above. Comparative simulations were then performed for the C450A-mutated AsLOV2 domain.

The Caver 3.0 program^45^ has been used for analysis of possible transport tunnels in all protein structures downloaded from the RCSB protein database as well as all structures saved from MD simulations.

The BIOVIA Discovery Studio^46^ visualizer was also used for complementary visualization and analysis of the modeled molecular structures.

### Top-down mass spectrometry

Proteins were desalted off-line on a Protein OptiTrap (Optimize Technologies, Oregon City, USA) with 0.1% formic acid (FA) in water and eluted with 80% acetonitrile/0.1% FA. Protein concentration were adjusted to 5 µM with water and the proteins were loaded into a quartz capillary ESI tip and mounted onto a home-built nESI source. Data were acquired in a broad band mode (m/z 200–3000) or in a CASI mode (Continuous Acquisition of Selected Ions) where selected charged states (19+, 20+, 21+ and 22+) of the protein were analyzed and fragmented simultaneously. Protein fragmentation was done through collision-induced dissociation in the quadrupole (front end) of 15 T FT-ICR MS (solariX XR, Bruker Daltonics, Bremen, Germany). Data were interpreted by a software tool developed in the laboratory and validated manually in Data Analysis 4.1 (Bruker Daltonics).

### Bottom-up mass spectrometry

Samples (10 µg) of each wild-type and C450A AsLOV2, both irradiated and non-irradiated, were digested using trypsin or Asp-N in 100 mM Tris-HCl pH 8.5 at 37 °C overnight. Subsequently, the samples were analyzed using LC-MS/MS. Peptides were injected on a reversed phase trap column (Zorbax 300SB-C18 5 μm, 0.3 × 5 mm, Agilent Technologies, USA) and desalted by 0.1% FA in water for 3 min at flow rate of 20 µL/min. Next, the peptides were eluted and separated on an analytical column (ZORBAX 300SB-C18, 0.3 × 150 mm, 3.5 µm, Agilent, USA) using a linear acetonitrile gradient from 5 to 35% of solvent B. Solvents were as follows: A: 0.1% FA in water, B: 0.1% FA, 95% ACN in water. The flow on the analytical column was 10 µL/min and the temperature was held at 50 °C. Eluting peptides were analyzed online with ESI-FT-ICR MS (15 T solariX XR, Bruker Daltonics, Germany) operating in data-dependent mode. The data were processed by DataAnalysis 4.1 (Bruker Daltonics, USA) and then searched by MASCOT in ProteinScape 4 (Bruker Daltonics, USA) against a database containing the AsLOV2 wt and C450A sequences. Various oxidative modifications of Met, Phe, His, Trp, Tyr, Pro and Cys were set as a variable. Parallel analysis in PEAKS X Studio was also conducted with automatic search for possible modifications present in the Unimod database. All found oxidized peptides and their non-oxidized variants were then manually searched in the data and their extracted ion chromatograms and highest intensity monoisotopic peaks were compared to estimate the level of oxidation. Assignment of oxidative modification to specific amino acids was done based on the fragment ions in the MS/MS spectra.

## Results

In this work, singlet oxygen is generated through the interaction of triplet state FMN (^3^FMN) with molecular oxygen. The kinetics of the ^3^FMN transient absorption signals and ^1^O_2_ phosphorescence were analyzed considering two assumptions. First, the small size of the studied AsLOV2 proteins (~3.5 nm diameter) suggests that ^1^O_2_ diffuses out of the protein matrix relatively fast. Due to this fact, ^1^O_2_ spends most of its lifetime in the water environment. The justification of this assumption is given in the Supporting information.

The second assumption is related to the fact that the FMN molecule is released from the protein to solvent due to oxidative modifications of the protein. The FMN released to water leads to an increase of ^1^O_2_ production outside of the protein. The presence of FMN in the aqueous environment was also observed in the work of Westberg *et al*.^23^ at low irradiation. In this work, we found evidence that FMN is present in the aqueous environment and its amount in the solution increases with irradiation of the sample.

### Determination of ^3^FMN lifetime

**Figure 1:**
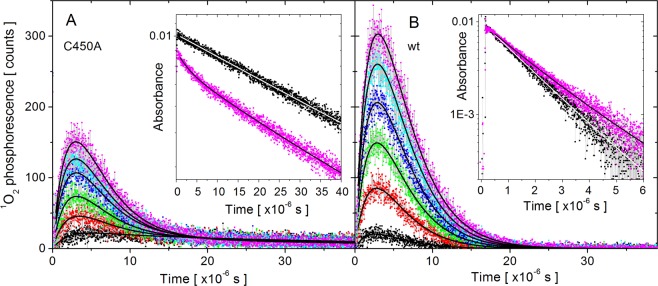
The time-course of the singlet oxygen phosphorescence signal for AsLOV2 C450A (**A**) and AsLOV2 wt (**B**) at different irradiation levels. The six curves were fitted in a global fitting procedure; the lines represent the fitted results. Insets: The decay of the FMN triplet state absorbance at 633 nm following the excitation laser pulse as measured for AsLOV2 C450A and AsLOV2 wt. Black and magenta points correspond to low and high irradiation, respectively.

The time-resolved ^3^FMN absorbance at 633 nm of the AsLOV2 C450A and the AsLOV2 wt are shown in insets of Fig. 1. The obtained ^3^FMN lifetime values for both AsLOV2 variants are summarized in Table 1. The black points belong to low irradiation (obtained during the first 12.5 min of the irradiation), while the data taken after extensive irradiation (75 min) of the samples are shown in magenta.

**Table 1:** The lifetime values of ^3^FMN and ^1^O_2_ as determined by fitting the time-resolved absorbance and phosphorescence data.

	AsLOV2 C450A		AsLOV2 wt	
	low irradiation (12.5 min)	high irradiation (75 min)	low irradiation (12.5 min)	high irradiation (75 min)
τ_T_^prot^ [µs]	57 ± 2		1.5 ± 0.01	
τ_T_^prot*^ [µs]	32 ± 7	23 ± 3	—	—
τ_T_^water^ [µs]	2.7^F^		2.7^F^	
τ_Δ_ [µs]	2.7 ± 0.2	3.0 ± 0.2	2.6 ± 0.2	3.3 ± 0.2

^F^fixed value, not varied during the fitting procedure.

### Singlet oxygen phosphorescence

The experimental time-courses of the ^1^O_2_ phosphorescence signals are shown in Fig. 1A,B for the AsLOV2 C450A and the AsLOV2 wt, respectively. The phosphorescence kinetics changes dramatically upon prolonged irradiation of the samples. For both AsLOV2 variants, the intensity of the phosphorescence signal increased significantly after 75 min of irradiation, indicating enhanced ^1^O_2_ production. Analysis of the ^1^O_2_ phosphorescence signals relies on the ^3^FMN lifetime values, *τ*_T,i_, determined in the flash photolysis experiments. For all the six irradiation levels, the time-dependence of singlet oxygen phosphorescence *P*(*t*) was assumed to consist of independent contributions, which correspond to different ^3^FMN groups:

1 $${P}({t})=\sum _{{i}}[\frac{{{A}}_{{i}}{{\tau }}_{\varDelta }}{{{\tau }}_{\varDelta }-{{\tau }}_{{\rm{T}},{\rm{i}}}}({{e}}^{-\frac{{t}}{{{\tau }}_{\varDelta }}}-{{e}}^{-\frac{{t}}{{{\tau }}_{{\rm{T}},{\rm{i}}}}})]$$

where *A*_i_ is amplitude of the phosphorescence and *τ*_Δ_ is the lifetime of the ^1^O_2_ molecule.

In agreement with the assumption that singlet oxygen, wherever being produced, spends most of its lifetime in the aqueous environment, only a single lifetime of singlet oxygen, τ_Δ_, was assigned to all the different contributions. It is noted that this approximation holds true due to the relatively short singlet oxygen lifetime in water environment and low protein concentration.

In the following sections, detailed analyses of ^3^FMN lifetimes and ^1^O_2_ phosphorescence are presented for the AsLOV2 C450A and AsLOV2 wt.

### AsLOV2 C450A. The ^3^FMN lifetime

Based on the transient absorption signals measured with the AsLOV2 C450A, we concluded that three different FMN groups are present in the system. These groups were assigned to the FMN inside the intact protein, the FMN inside the oxidized protein and the FMN in the water environment. The corresponding ^3^FMN lifetimes (τ_T_^prot^, τ_T_^prot*^, and τ_T_^water^) were determined by fitting all the 18 decay curves with triple-exponential decays using the following constrictions: τ_T_^prot^ - a single fitting parameter used for all the decay curves; τ_T_^prot*^ - no restrictions, this lifetime was allowed to evolve during the irradiation; τ_T_^water^ - not fitted, fixed to 2.7 µs - the lifetime measured by our apparatus in aqueous solution of FMN, which is in agreement with previously reported value^2^.

The ^3^FMN lifetime values obtained for AsLOV2 C450A can be summarized as follows: τ_T_^prot^ = 57 ± 2 µs; τ_T_^prot*^ - gradually decreases from 32 ± 7 µs (at low-irradiation) to 23 ± 3 µs (at high-irradiation) (Figure S1). The decreasing τ_T_^prot*^ lifetime is likely caused by the gradually increasing accessibility of the protein interior to oxygen due to by the irradiation-induced oxidation. The ^3^FMN lifetime determined for AsLOV2 C450A, τ_T_^prot^ = 57 µs, is in accordance with the corresponding lifetime values previously reported for the C450A variant, i.e. 72 µs^31^ and 98 µs^47^, and also with the lifetimes found in miniSOG, τ_T_^prot^ = 28 µs, and SOPP, τ_T_^prot^ = 79 µs at 30 ^o^C^26^.

**Figure 2:**
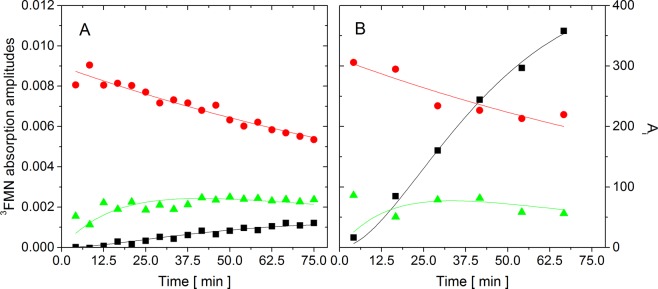
The amplitudes of the exponential ^3^FMN absorption decays, Abs_i_^0^ (**A**) and the amplitudes A_i_ of the different ^3^FMN group contributions to the ^1^O_2_ phosphorescence signal (see Eq. 1) (**B**) in intact protein (red circles), in oxidized protein (green triangles), and in water (black squares) as a function of the irradiation time of AsLOV2 C450A. The solid lines represent the results of the fitting procedures.

Additional information can be derived from the amplitudes Abs_i_^0^ of the triple-exponential decay curves (Σ_i_Abs_i_^0^exp(−t/τ_Ti_)), which represents ^3^FMN absorption right after the excitation laser pulse (Fig. 2A). The direct comparison of the initial absorption amplitudes is difficult because of the unknown extinction coefficients for all three different ^3^FMN groups. Despite this drawback, the absorption amplitudes provide an important information about the kinetics of the system evolution.

**Scheme 1:**
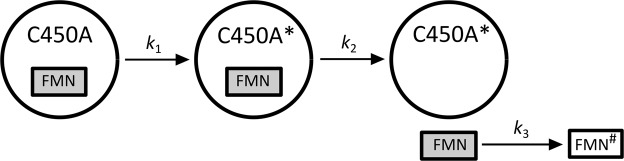
The scheme of the irradiation-induced changes in AsLOV2 C450A and FMN dissociation. C450A and C450A* represent the intact and the oxidized protein, respectively. FMN and FMN^#^ represent the intact and the bleached flavin cofactor, respectively.

Based on the experimental observation, we proposed a model that schematically describes irradiation-induced changes in AsLOV2 C450A accompanied by FMN release (Scheme 1).

The protein oxidation is characterized by the unimolecular rate constant *k*_1_. The constant *k*_2_ represents the rate constant of the FMN release from the oxidized protein. For the sake of simplicity, only a single unimolecular rate constant *k*_2_ was used for all the proteins with different level of oxidation. Finally, the bleaching of FMN in the water environment (rate constant *k*_3_) was taken into account in the model. Precise description of the model by differential equations and the corresponding analytical solutions for different FMN groups’ concentrations [FMN]_i_ are shown in Supporting information. It is noted that the values of *k*_1_, *k*_2_, and *k*_3_ depend on the irradiation conditions and are specific for the present experiment.

To determine the rate constant of FMN bleaching, *k*_3_, in the solution, we performed separate experiments. For this purpose, 25 µM FMN was mixed with 25 µM apoAsLOV2, which was prepared according to the procedure of Dürr *et al*.^48^ and was irradiated in the same way as both AsLOV2 variants. Noteworthy, upon admixture of apoAsLOV2 and FMN, formation of holoAsLOV2 was not detected based on UV-Vis absorbance measurement. The apoAsLOV2 wt was used to mimic the presence of the protein in the solution. As expected, significant bleaching of FMN was observed and both the production of singlet oxygen and the initial absorbance values Abs^0^_water_ of ^3^FMN decreased exponentially. The rate of the bleaching was determined as 1/*k*_3_ = 3260 s, which reflects both the decay of ^1^O_2_ production, and the decay of the ^3^FMN absorption amplitudes (Abs^0^_water_). The ^3^FMN lifetime remained unaffected, 2.7 ± 0.1 µs, during the irradiation experiment.

The obtained kinetics of ^3^FMN absorption amplitudes (Fig. 2A) for AsLOV2 C450A were fitted according to the model shown in Scheme 1 (assuming proportionality between: (i) the concentration of FMN, (ii) the amount of ^3^FMN produced and (iii) the ^3^FMN absorption signal Abs_i_^0^ in each group, throughout the irradiation experiment). The data points of Abs^0^_prot_ were fitted by the single exponential decay (Eq. S1), from which the characteristic time, 1/*k*_1_ = 9000 s was obtained for the rate of protein oxidation in our system. The value of *k*_2_ was determined by fitting the ^3^FMN absorption in the oxidized protein Abs^0^_prot*_ and in the solution Abs^0^_water_ with the corresponding time-dependences (Eqs. S2 and S3), using the previous values of *k*_1_ and *k*_3_. The best match of the experimental data and the analytical curves was obtained for 1/*k*_2_ = 1000 s. Based on the fits shown in Fig. 2A, we can conclude that the experimental data are well described by the model.

### AsLOV2 C450A. Singlet oxygen phosphorescence

The ^3^FMN lifetime values (τ_T_^prot^, τ_T_^prot*^, and τ_T_^water^) were utilized to analyze the ^1^O_2_ phosphorescence data. The phosphorescence amplitudes A_prot_, A_prot*_ and A_water_ and the lifetime of singlet oxygen τ_Δ_ were determined in a global fit (using all the measured curves shown in Fig. 1A). The lifetime of ^1^O_2_ was allowed to evolve during the irradiation. The fitted curves and the obtained amplitudes are shown in Figs. 1A and 2B, respectively. The evolutions of A_water_ and A_prot_ were fitted with the corresponding time-dependences of [FMN]_water_ and [FMN]_prot._, respectively. The general shape of these curves reproduces the experimental data very well. Based on the proposed model, the proportionality factors of the two curves (black and red line in Fig. 2B) can be used to calculate the quantum yield of singlet oxygen production in the intact AsLOV2 C450A, using the quantum yield of ^1^O_2_ production by FMN in the solution (Φ_Δ water_ = 0.57)^2^ as a reference. Taking into account the different absorbance of the two FMN groups at the excitation wavelength (475 nm) (Figure S2), the Φ_Δ_ value for the intact AsLOV2 C450A equals to 0.07. The analogous quantitative analysis of the A_prot*_ data is not feasible due to the changing level of oxidation during the experiment.

### AsLOV2 wt. The ^3^FMN lifetime

The kinetics of ^3^FMN transient absorption in the AsLOV2 wt (inset Fig. 1B), were analyzed in analogous way as for the AsLOV2 C450A variant. In the case of the AsLOV2 wt, only two triplet state lifetime components were identified. Based on this observation, we concluded that FMN is released from the oxidized AsLOV2 wt very fast. This is in agreement with our results from molecular dynamics studies, which indicate a steric clash between the oxidized Cys450 and FMN (see below). The ^3^FMN absorption decays were fitted with two lifetime values (τ_T_^prot^, τ_T_^water^), assuming that these lifetimes do not change during the irradiation: τ_T_^prot^ - a single fitting parameter used for all the decay curves; τ_T_^water^ - not fitted, fixed to 2.7 µs, which is the lifetime measured in pure FMN solution. The global fit resulted in a value of τ_T_^prot^ = 1.50 ± 0.01 µs. The low error indicates high-quality fits.

The obtained lifetime of the ^3^FMN state in AsLOV2 wt is in good agreement with the results of Swartz *et al*., τ_T_^prot^ = 2 µs^31^ and Song *et al*., τ_T_^prot^ = 2.2 µs^49^, but differs from the triplet state decay time reported by Gil *et al*., τ_T_^prot^ = 9.5 µs^47^.

**Figure 3:**
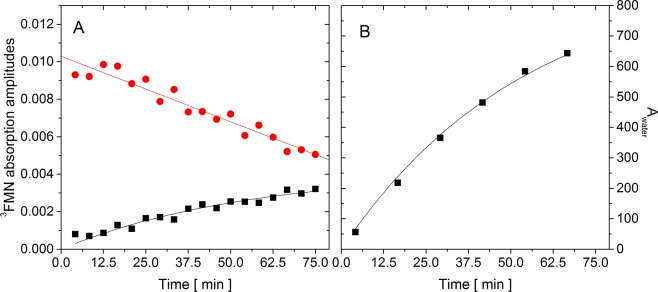
The amplitudes of the exponential ^3^FMN absorption decays, Abs_i_^0^ (**A**) and the amplitudes A_i_ of the different ^3^FMN group contributions to the ^1^O_2_ phosphorescence signal (see Eq. 1) (**B**) in intact protein (red circles) and in water (black squares) as a function of the irradiation time of AsLOV2 wt. The solid lines represent the results of the fitting procedures.

**Scheme 2:**
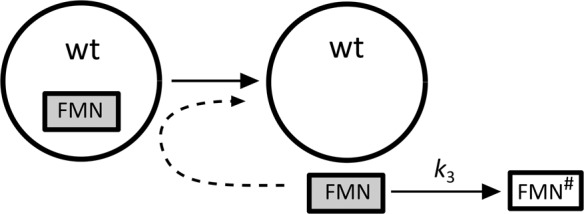
The scheme of the irradiation-induced changes in AsLOV2 wt and FMN dissociation. FMN and FMN^#^ represent the intact and the bleached flavin cofactor, respectively.

The amplitude of the ^3^FMN absorption Abs^0^_prot_ indicates that the amount of ^3^FMN produced in the intact protein (Fig. 3A, red points) decreased by 50%. At the same time, there is no evidence of slowing the decrease down towards longer irradiations. In this case, the experimental data are better fitted with a linear decrease than with an exponential decay. This behavior is consistent with the assumption of the fast FMN release from the protein and can be rationalized as follows: the quantum yield of ^1^O_2_ production is very low for the FMN inside the AsLOV2 wt. Once the FMN is released from the protein, the rate of ^1^O_2_ production increases, which in turn enhances the rate of the protein oxidation and the rate of FMN release. In principle, the FMN release acts as an auto-catalyzed reaction, which explains the unusual (quasi-linear) decrease of Abs^0^_prot_. Scheme 2 shows a model of irradiation-induced changes in the AsLOV wt accompanied by FMN release.

The mathematical description of this model is shown in the Supporting information. This model was used to fit the Abs_i_^0^ values in Fig. 3A. The curvature of the Abs_water_ (black squares), reflecting the bleaching effect, is well described by the rate constant *k*_3_.

### AsLOV2 wt. Singlet oxygen phosphorescence

The procedure of the analysis of ^1^O_2_ phosphorescence data of the AsLOV2 wt were analogous as in the case of AsLOV2 C450A, taking into account the different model of irradiation-induced changes in the protein. Based on the fitting results, we conclude that the observed phosphorescence signal in AsLOV2 wt can be fully explained by ^1^O_2_ production in the solution and the contribution of the FMN_prot_ group can be considered as negligible. The results of the phosphorescence global fits and the obtained amplitudes A_water_ are shown in Figs. 1B and 3B, respectively. The A_water_ values are well fitted with the [FMN]_water_ time-dependence using the same value of 1/*k*_3_ = 3260 s.

**Figure 4:**
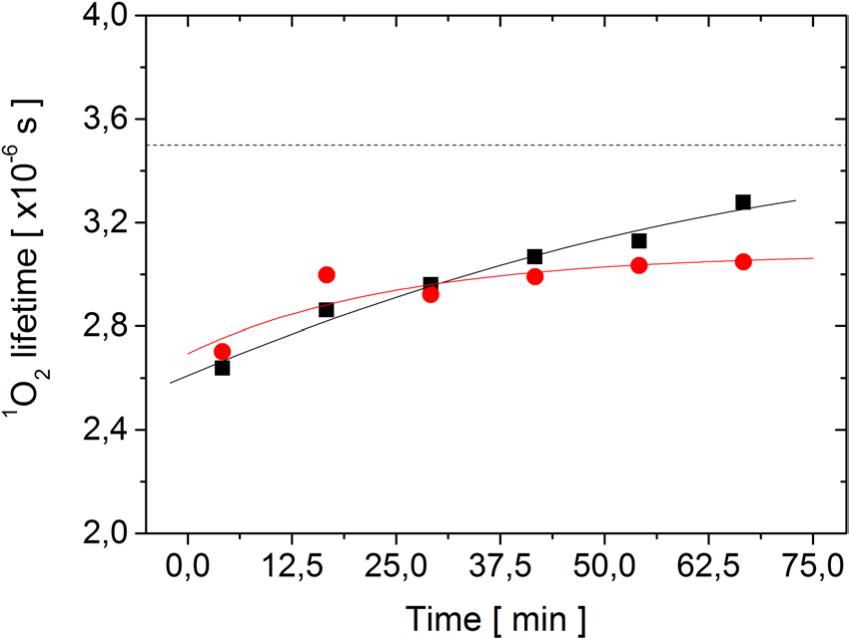
Dependence of the ^1^O_2_ lifetime on irradiation time in AsLOV2 C450A (red circles) and AsLOV2 wt (black square). The estimated error of individual data points is ±0.2 μs. The solid lines serves as an eye lead. The dashed line is the ^1^O_2_ lifetime in pure water^70^.

The global fit (based on the developed model) allowed us to determine the dependence of ^1^O_2_ lifetime on irradiation time (Fig. 4). The results show than in both AsLOV2 variants the lifetime of ^1^O_2_ increases with irradiation time very likely as a result of progressive proteins oxidation.

### Changes in AsLOV2 optical spectra induced by irradiation

#### UV-VIS absorption spectroscopy

UV-VIS absorption spectra of the wild type and the C450A variant of AsLOV2 are almost identical in the spectral range 300–550 nm and correspond to the absorption of FMN (Figure S2). Both variants exhibit major absorption peaks at 447 nm, 473 nm, and a shoulder at ~425 nm. The difference between these two forms in the region 300–400 nm reflects the replacement of cysteine for alanine in position 450^31,32^. After blue light irradiation, a small spectral change in the region 400–500 nm is observed in both AsLOV2 forms, corresponding to ~12% and ~22% decrease in the absorbance at 447 nm for AsLOV2 C450A and AsLOV2 wt, respectively (Figure S3). The observed decrease is usually attributed to chromophore bleaching. The small increase in the absorbance in the region 300–400 nm has been previously interpreted as a result of tryptophan oxygenation to kynurenine^50^, which is in accordance with the results obtained by mass spectrometry (see below).

#### Circular dichroism

Structural changes in AsLOV2 proteins induced by irradiation were also followed by circular dichroism (CD) spectroscopy in the far- and near- UV regions (Figure S4). In the far-UV region, the CD spectra show that both variants of AsLOV2 contain similar fractions of α-helical (~13 ± 3%) and β-sheet (32 ± 3%) structures (for the analysis we used two different web servers: DICHROWEB^51,52^ and BeStSel^53^). Small changes in the secondary structure in both variants of AsLOV2 are noticeable after the extensive laser irradiation (Figures S4A,C), corresponding to ~13% and ~25% decrease in ellipticity at 222 nm for AsLOV2 C450A and AsLOV2 wt, respectively. The extent of the changes is similar both in the far-UV and the near-UV regions to those observed in the corresponding UV-VIS absorption spectra. The observed changes can be attributed to a direct effect of ROS or can be of a secondary nature as a result of the chromophore oxygenation and/or its release^54^.

#### Fluorescence

**Figure 5:**
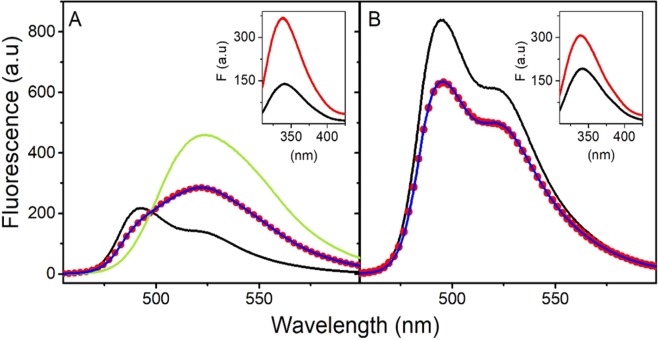
Fluorescence emission spectra of FMN and intrinsic tryptophan in AsLOV2 wt and AsLOV C450A. The fluorescence of FMN in AsLOV2 wt (**A**) and in AsLOV2 C450A (**B**) before (black line) and after (red dots) illumination and the fluorescence of free FMN (green). The blue line shows the fit to FMN fluorescence after the illumination. The fits (blue line) consist of two fractions of fluorescence: protein-bound and free FMN. Insets: Intrinsic tryptophan fluorescence of the corresponding forms of AsLOV2 before (black line) and after illumination (red line). The concentrations of proteins and the free FMN were 10 µM in 20 mM Tris-HCl, pH 7.8.

AsLOV2 contains two intrinsic fluorescent probes, FMN chromophore and one tryptophan residue, Trp491. Comparison of the fluorescence emission spectra of the flavin chromophore located in the AsLOV2 wt and its variant AsLOV2 C450A clearly shows significantly lower intensity of the fluorescence in the AsLOV2 wt (Fig. 5). This is due to light-induced formation of covalent bond between flavin chromophore and the reactive cysteine in the AsLOV2 wt. The fluorescence maximum of the wild-type protein after irradiation is moved to higher wavelengths, corresponding to the peak of free FMN at 520 nm (Fig. 5A). These results suggest that part of the flavin chromophores are released from the binding pocket into the solvent. In fact, we fitted the fluorescence spectrum of AsLOV2 wt after irradiation as a combination of the fluorescence spectra of protein bound FMN and free FMN. The obtained fit consists of two fractions combining ~45% of the free FMN and ~55% protein-bound FMN (Fig. 5A). In the case of AsLOV2 C450A, the irradiation induces ~23% decrease in fluorescence intensity measured at 500 nm (Fig. 5B). An analogous fit for AsLOV2 C450A (Fig. 5B) led to fractions of free and protein-bound FMN equal to ~25% and ~75%, respectively. On the other hand, the intrinsic tryptophan fluorescence of AsLOV2 C450A before irradiation is ~20% higher than the fluorescence of AsLOV2 wt (insets Fig. 5). The irradiation induces ~1.6-fold and ~2.6-fold increase in fluorescence intensity of AsLOV2 C450A and AsLOV2 wt, respectively. The absence of a shift in the maximum of the tryptophan fluorescence suggests that the observed irradiation-induced increase is not due to a conformational change in the proteins, but rather due to decreased fluorescence quenching by the flavin cofactor.

**Figure 6:**
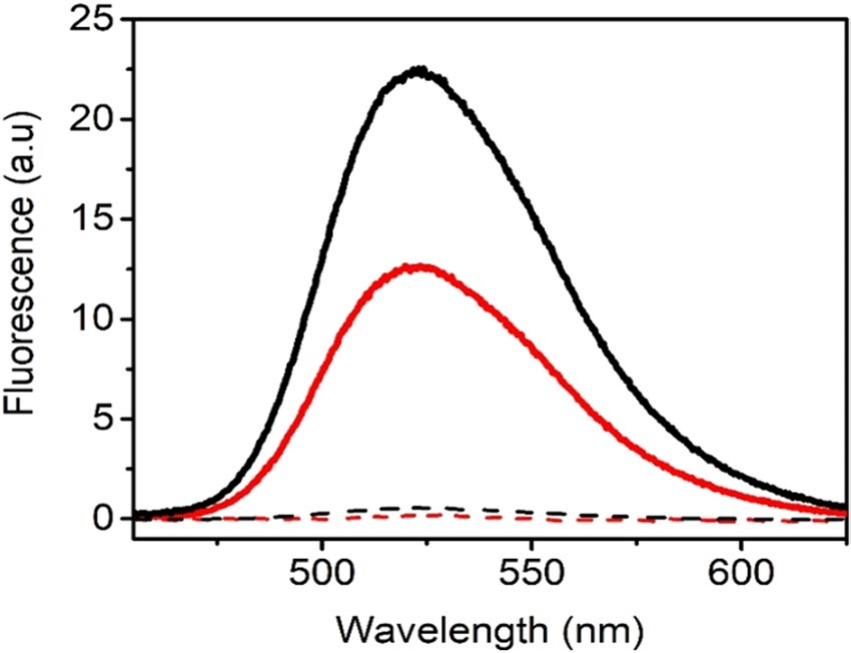
Analysis of a release of FMN from AsLOV2 wt and AsLOV2 C450A induced by blue light irradiation. FMN fluorescence indicates a release of FMN from its binding pocket to the bulk solvent. FMN fluorescence of non-irradiated AsLOV2 wt (black dashed), AsLOV2 C450A (red dashed) and irradiated AsLOV2 wt (black solid) and AsLOV2 C450A (red solid).

To address the possibility of FMN dissociation from the protein, we performed simple filtration experiments in which the released cofactor passes through the filter, while the protein is retained. The outcome of these experiments showed that before irradiation there was no free FMN detected in the filtrate. The irradiation-induced changes in the proteins led to release of FMN from both types of proteins (Fig. 6). Although this method does not allow quantitative determination of released FMN, it does allow a relative comparison: irradiation induced a release of ~1.8-fold more FMN in the case AsLOV2 wt in comparison with AsLOV2 C450A variant (Fig. 6), which corresponds to the ratio of  dissociated FMN from AsLOV2 wt and AsLOV2 C450A obtained from the analysis of the fluorescence spectra in Fig. 5.

**Figure 7:**
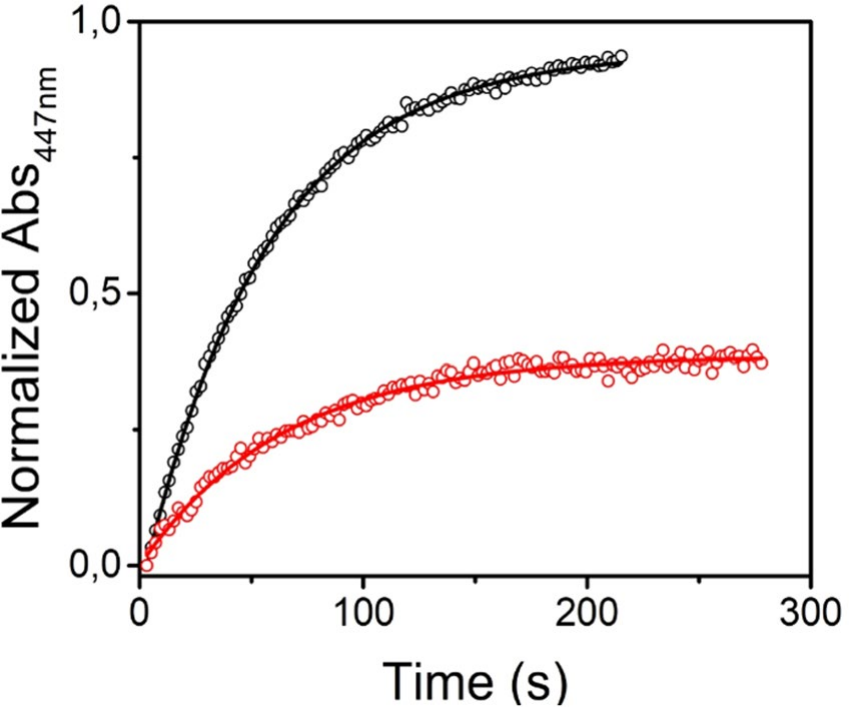
Dark state recovery kinetics of non-irradiated (black) and irradiated (red) AsLOV2 wt. Experimental data are shown as circles and the solid lines show fits of the data by single exponential functions.

Another way to assess the fraction of retained FMN in the protein after irradiation is to compare the amplitude of the cysteinyl-FMN adduct formation in the AsLOV2 wt measured by flash-induced absorbance changes at 447 nm^55^ using samples before and after the extensive (75 min) irradiation by laser (Fig. 7). The obtained results suggest that after irradiation only ~40% of FMN is able to form the adduct.

### Determination of irradiation-induced changes in primary structures of AsLOV2

Light-induced production of ^1^O_2_ by FMN is accompanied by covalent modification of amino acids in AsLOV2, which localization and nature of the modification were determined. First, we measured the intact mass to verify the protein state and possible fragmentation. Based on these data (Figure S5A), we concluded that the irradiated proteins are intact, no significant fragmentation occurred but they differ in the extent of oxidative modifications, with C450A form being significantly more affected upon irradiation (Figure S5B).

**Figure 8:**
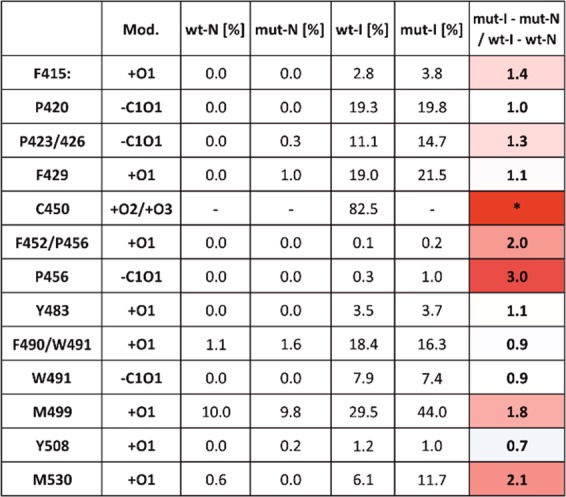
List of oxidation products and their extent of oxidation (intensity of oxidatively modified peptide) in all studied protein forms – wild-type (wt) and C450A (mut) before (N) and after (I) irradiation. Column Mod shows the elemental composition of the modification (oxidation: +O1, +O2 or +O3; Trp to kynurenin: -C1+O1; Pro to pyrrolidone: -C1O1). When it was not possible to clearly assign oxidation to one specific residue, two possibly affected residues are listed in the first column. The last column highlights the fold change in oxidation between C450A and wt forms. In case of C450 oxidation (marked in the last column with an asterisk) such comparisons were not possible due to generation of different peptides upon oxidation and due to mutation. The intensity of oxidized form was indirectly deduced from intensity decrease of the intact, unoxidized Asp-N generated peptide.

In the bottom-up approach, the protein was digested in solution, analyzed by LC-MS/MS and the generated peptides were identified by database searching and automated *de novo* sequencing. This yielded a list of modified residues and allowed a direct comparison between the different AsLOV2 states regarding the extent of oxidation of individual amino acids (Fig. 8). By this approach, 97% coverage was achieved and only five residues (460–464) were missed (Figure S6). Observed oxidation of the His-tag sequence (N-terminal Met and histidines) are not listed among the modified residues in Fig. 8, as this part of the sequence (the first 31 amino acids) is unnatural for AsLOV2 (Figure S6).

In the AsLOV2 wt, di- and tri- oxidation of Cys450, (but not single oxidation) were found to be very prominent modifications. While the bottom-up method targeted the whole bulk of protein populations, the top-down approach allowed the selection of the “first-hit”/singly oxidized species, and their subsequent fragmentation and assignment of modified residues based on the protein MS/MS spectra. The selection of singly oxidized protein forms had also another benefit as it “filtered-out” the Cys450 modification, which occurred as double and triple oxidation and allowed us to focus on other residues. Unfortunately, the gas-phase fragmentation of AsLOV2 proved to be quite inefficient as we missed fragmentation in the middle of the protein (Figure S7A). Nonetheless, we obtained the information supporting and complementing our bottom-up data. Based on the oxidation increase between the N-terminal fragment ions b26 and b47 together with the same trend observed from the opposite site (C-terminal fragment ions y127 and y151) we can point on Phe415 as an oxidation-sensitive amino acid. Furthermore, we can also conclude (based on fragment ion y36, Figure S7B), that the sequence spanning from Gly511 to the C-terminus is not affected by oxidative events in any of the analyzed AsLOV2 forms. This is at first in apparent contrast with the bottom-up data, where Met530 was found to be oxidized and where the data point to a significant difference between wt and C450A forms (Fig. 8). However, one should keep in mind that the top-down fragmentation was aimed at the first-hit oxidation and thus we can assume that Met530 oxidation is a secondary event. Finally, we can state that the remaining primary oxidation sites are localized between amino acids 420 and 511 where the majority of significantly oxidized residues were found by the bottom-up approach.

Altogether, based on the MS data it can be concluded that: (i) the modifications occurring in AsLOV2 upon irradiation consist of various products of amino acid side chain oxidations, (ii) the modifications are light-dependent as the overall extent of the oxidative modifications is much higher in irradiated samples of both AsLOV2 wt and the AsLOV2 C450A variant, (iii) generally the same amino acids are oxidized in both AsLOV2 variants, (iv) the extent of some oxidative modification is higher in AsLOV2 C450A compared to AsLOV2 wt.

### Detection of tunnels in the structure of AsLOV2

**Figure 9:**
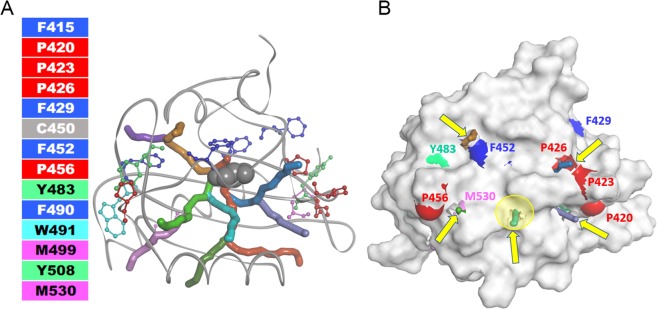
Discovery Studio visualization of Caver-calculated tunnels of minimized PDB ID: 2v0u structure. (**A**) Ribbon representation of the protein with color-coded amino acids (summarized in Fig. 6) shown in ball and stick representation. C450 is shown in grey CPK representation. The color coding of the tunnels represented as CPK does not correspond to the color coding of the amino acids and is for their distinction only. (**B**) Surface representation of the protein with color coding of the amino acids of interest on the surface. The yellow arrows highlight the tunnels reaching the protein surface. The ribityl chain of FMN structure (not shown) overlaps with the circled tunnel 1 (yellow semitransparent circle). Detailed information from 5 ns MD simulations is summarized in Figure S9.

The unusual modification of proline residues (highlighted in red in Figs. 9 and S8, illustrating that P420, P423 and P426 form a cluster on the right side of the protein) led us to an assumption that in this part of the protein exit channel(s) may exist, connecting the source of the production of ^1^O_2_ with the protein surface. As a result, even prolines, which are usually resistant to redox reactions, are oxidized by reactive oxygen species created by FMN. Indeed, the Caver 3.0^45^ tunnel analysis of AsLOV2 wt crystal structure (PDB ID: 2v0u) detected the presence of several tunnels. We have analyzed several AsLOV2-related protein structures: at first we considered their optimized geometries and then their geometries resulting from 5 ns MD simulation in water environment: (i) dark state of AsLOV2 wt (PDB ID: 2v0u), i.e. without covalent bond between C450 and C4(a) of the isoalloxazine ring (Figure S8A), (ii) *in silico* SOO^−^ substitution on C450 Sulphur (Figure S8B), (iii) light state of AsLOV2 wt (PDB ID: 2v0w), i.e. with covalent bond between C450 and C4(a) of the isoalloxazine ring (Figure S8C), and (iv) *in silico* mutated AsLOV2 C450A (Figure S8D). The presence of tunnels was detected in all the protein geometries (Figures S8A–D). However, the number of tunnel clusters, as indicated on the pie chart on Figure S8 slightly differs, related to the C450 modifications/FMN binding. Several tunnel clusters (channels) (Figs. 9 and S8) are believed to be lined by the above mentioned P420, P423 and P426 amino acids. Several other channels, as illustrated in Figs. 9 and S8 terminate in the proximity of the highlighted amino acids listed in Fig. 8 and are shown as CPK representation in Figure S8. Interestingly, the comparison of a number of channels observed in MD simulations in AsLOV2 wt and AsLOV2 C450A suggests that C450A exhibits slightly more tunnels and could thus suffer a larger damage by oxygen than AsLOV2 wt. The highest number of calculated tunnels was found in 2v0u-SOO^−^, which in the light of our results might point to increased dynamics of the protein matrix explaining thus more efficient release of FMN from the protein.

## Discussion

The protein matrix surrounding FMN can efficiently decrease the Φ_Δ_ value by quenching the excited triplet state of FMN and by restriction of oxygen diffusion towards the isoalloxazine ring^56^. This, on one side, may have a protective role for the photosensitizers close surrounding by preventing unwanted production of ROS but on the other hand, significantly limits the ROS effect on the site of an intendent action. Here, we argue that genetically-encoded photosensitizers may be used as a carrier of the reactive cofactor, and the release of the cofactor can be modulated by an irradiation or possibly other perturbation of the protein matrix.

### Primary structure modification of AsLOV2 upon irradiation

As can be deduced from the fine structure of FMN absorption spectrum in the wavelength range 400–500 nm, the cofactor has no extensive contacts with solvent molecules^57^. This is also in accordance with the crystal structures of the closely related flavoproteins SOPP and miniSOG^2^. On the other hand, our *in silico* analysis of the AsLOV2 crystal structure^58^ suggests the existence of several channels in the protein structure, connecting the reactive site C(4a) on the isoalloxazine ring with the protein surface (Fig. 9). This connection becomes even more obvious after molecular dynamics simulation (Figure S8). The presence of channels was also identified by molecular dynamics simulation in the case of miniSOG^59^. If the channels play an active role as a traffic route of molecular oxygen to the FMN binding site and diffusion of the ^1^O_2_ and possibly other ROS out to the protein surface, one can expect oxidatively modified amino acids localized close to these channels. Indeed, the localization of three modified prolines, Pro420, Pro423, and Pro426, at the orifice of a cluster of several channels provide an explanation of unexpected modification of this otherwise very oxidation-resistant amino acid (Fig. 9). Our results strongly support the ability of reactive oxygen species to react with proline^60,61^. Because some reports conclude that proline does not interact with ^1^O_2_ our observations suggest a production of other ROS than ^1^O_2_ by FMN when encapsulated in AsLOV2^62–64^.

ROS effectively react with aromatic amino acids such as Trp and Tyr. One surprising observation regarding oxidative modification of amino acids in AsLOV2 was that none of the three tyrosine residues, present in AsLOV2, were modified by ROS produced by FMN. There are two ROS species that can be produced by FMN, O_2_^•−^ and ^1^O_2_. While O_2_^•−^ is relatively unreactive and directly reacts only at a few specific protein sites^65^, ^1^O_2_ reacts efficiently with five common and functionally important amino acids (Cys, His, Met, Trp, Tyr)^5,8,66^. However, our findings show that ROS produced by AsLOV2 variants, surprisingly, do not oxidize the tyrosine residues of AsLOV2. Noteworthy, the absence of modification of tyrosine residues by ^1^O_2_ was also noticed in the case of two unrelated proteins such as cytochrome *c* (containing 4 Tyr residues)^67^ and lysozyme (containing 3 Tyr residues)^68^.

The absence of the irradiation-induced modification of electron rich amino acids such as Tyr and Phe in both variants of AsLOV2 is unexpected on chemical grounds. Kiselar *et al*.^69^ speculated that some of the oxidative modification of aromatic residues in general can be “transferred” to Met by radical transfer. This suggestion could help explain the rather significant increase in Met oxidation, but only low Tyr and Phe oxidation in C450A.

A higher number of oxidative modifications of amino acids detected in our work in comparison with a very recent paper by Torra *et al*.^29^ can be attributed to the differences in the solution irradiation (wavelength, power, etc.) and partially also to the higher sensitivity and resolution obtained by our LC-MS/MS analysis on FT-ICR MS and combination of proteomics approaches directed at localization of the modification sites. Our approach truly analyzes all protein states present in the sample and, in addition, the top-down analysis allows the identification of the first-hit residues. In contrast, Torra *et al*.^29^ identified the oxidized residues in the crystal structure, hence the possibility that they observed just the crystallization-capable population cannot be excluded.

### Approaches to increase singlet oxygen production by LOV domains

The models based on our data demonstrate the fact that irradiation of both AsLOV2 variants is accompanied by FMN dissociation from the protein matrix. However, the dissociation as well as the production of ^1^O_2_ is more efficient in AsLOV2 wt than in AsLOV2 C450A. The observed increase in ^1^O_2_ yield is thus rather the consequence of FMN release than of the protein oxidation.

In several previous studies, the authors assumed that an increase in ^1^O_2_ production was due to blocking concurrent reactions by electron transfer from redox-active amino acids such as Tyr, Phe, Met, Trp, and Cys, which quench the triplet state of FMN and may thus lead to formation of O_2_^•−^^29^. In fact, this assumption led to efforts to rationally design genetically encoded efficient flavoproteins by replacing amino acids responsible for quenching of the FMN triplet state^23,26^, allowing thus increased production of ^1^O_2_. These efforts indeed led to an increased Φ_Δ_ value attained in miniSOG and SOPP without detecting a dissociation of FMN^21,26^. These observations suggest that ^1^O_2_ production in genetically-encoded photosensitizers can be achieved also without a release of the cofactor from the protein matrix.

### Impact of mutation C450A on FMN release

The combined analyses of the data obtained from ^3^FMN absorption experiments (Fig. 3A), from the analytical models as well as from fitting of irradiation-induced FMN fluorescence of the free and protein-bound FMN (Fig. 5) point to the release of approximately 48 ± 3% and 28 ± 4% of FMN from AsLOV2 wt and AsLOV2 C450A, respectively, after irradiation of the proteins. About 1.8-fold higher release of FMN from AsLOV2 wt in comparison with AsLOV2 C450A was documented by direct determination of relative amount of FMN released from the irradiated proteins (Fig. 6). All these results suggest that AsLOV2 wt is more affected by irradiation than its variant C450A. Strikingly, the amplitude of irradiation-induced changes in absorbance and ellipticity (Figures S3 and S4) as well as the extent of changes detected by mass spectrometry analysis (Figure S7) clearly show the opposite, i.e. that the variant C450A is more perturbed than AsLOV2 wt. In fact, the analysis of the total (integrated) ^1^O_2_ phosphorence signal in both variants clearly shows that AsLOV C450A produced significantly higher amount of ^1^O_2_ (Figure S10) and likely also other ROS during irradiation, which explains the higher oxidation damage of AsLOV2 C450A in our experiments.

To reconcile our observations, we hypothesize that more efficient release of FMN from AsLOV2 wt than from its Ala-containing variant is due to irradiation-induced oxidation of Cys450. As illustrated in Figure S9, the “brute force” superposition of C450 and (per)oxidized C450 results in a steric clash of modified Cys450 and FMN. Molecular geometry optimization and the subsequent MD simulations can easily eliminate such inappropriate molecular contacts, but in real structures such intermolecular conflicts could facilitate the release of FMN from AsLOV2.

## Conclusions

We show that an irradiation-induced increase of ^1^O_2_ production in the AsLOV2 variants is due to a release of FMN to solvent as a result of oxidative modification of certain amino acids, predominantly the reactive cysteine 450, localized nearby the isoalloxazine ring in the AsLOV2 structure. Our findings may be utilized to design more efficient genetically encoded photosensitizers based on LOV domains. The protein scaffold can serve merely as a targetable carrier while the reactive cofactor would be released at the site of action by a suitable perturbation of the protein structure. In principle, intensive blue light irradiation or combined approach including both irradiation and thermogenesis could be applied. The irradiation can be more efficient in releasing FMN, if the binding site of isoalloxazine ring becomes repulsive upon irradiation, either through steric clashes or through charge repulsion. Enhanced effect, might be achieved by placing suitable amino acids close to the isoalloxazine ring. These amino acids, such as cysteine or methionine, upon irradiation-induced oxidation increase their volumes and form steric clashes, thereby repelling the flavin cofactor. Local thermogenesis could be an alternative approach that would increase FMN dissociation from AsLOV2, due to the increased dynamics of polypeptide chain and consequently the increase of the Φ_Δ_ value^2^.

## Supplementary information

Supplementary information
