# Supplementary material for: Photoinduced damage of AsLOV2 domain is accompanied by increased singlet oxygen production due to flavin dissociation
Source: Sci Rep. 2020 Mar 5;10:4119. doi: 10.1038/s41598-020-60861-2 (PMC7058016; doi:10.1038/s41598-020-60861-2)
Supplement: Supplementary file 1 — Supplementary information [file 41598_2020_60861_MOESM1_ESM.docx]

**Supporting information**

**to**

**Photoinduced damage of AsLOV2 domain is accompanied by increased singlet oxygen production due to flavin dissociation**

Martina Petrenčáková, František Filandr, Andrej Hovan, Ghazaleh Yassaghi, Petr Man, Tibor Kožár, Marc-Simon Schwer, Daniel Jancura, Andreas Plückthun, Petr Novák, Pavol Miškovský, Gregor Bánó and Erik Sedlák

**Predominant localization of singlet oxygen outside the protein**

Singlet oxygen produced inside the protein matrix diffuses to the surrounding water environment. An upper limit for the effective diffusion time of ^1^O_2_ escaping from the protein interior to water can be obtained using the analytical formula derived by Hovan et al. [Hovan et al. 2018]. Choosing reasonable limits for the ^1^O_2_ partition coefficient in the protein/water environment, *K_Δ_*<10, and for the diffusion constant of ^1^O_2_ inside of the protein, *D_Δ_*^prot^>1.10^-10^ cm^2^s^-1^ [Lepeshkevich et al. 2014], an upper estimate for the effective diffusion time, τ_Δ_^eff^<9 ns, was obtained for AsLOV2. This value is in good agreement with the estimated transient times reported for miniSOG and SOPP (3-60 ns) [Westberg et al. 2017]. The obtained escape-time was compared with the lifetime of ^1^O_2_ in the protein interior environment, which was estimated using the method suggested by Lepeshkevich et al. [2014] for globular proteins. The size of AsLOV2 and the rate constants for ^1^O_2_ deactivation by the individual amino acids (mostly Tyr, Trp, His, Met and Cys [Lindig et al. 1981, Michaeli et al.1994, Lepeshkevich et al. 2014]) were taken into account. The rate of ^1^O_2_ deactivation by FMN was estimated on the basis of data with free riboflavin [Wilkinson et al. 1995]. This way the lifetime of ^1^O_2_ was estimated to be τ_Δ_^LOV2^~400 ns for the AsLOV2 interior. This lifetime is significantly longer than the estimated escape-time. We therefore conclude that ^1^O_2_ produced in AsLOV2 spends most of its lifetime in an aqueous environment, which is in accordance with the assumption used by Westberg et al. [2017].

**References**

Hovan, A. *et al.* Phosphorescence Kinetics of Singlet Oxygen Produced by Photosensitization in Spherical Nanoparticles. Part I. Theory. *The journal of physical chemistry. B* **122**, 5147-5153, doi:10.1021/acs.jpcb.8b00658 (2018).

Lepeshkevich, S. V. *et al.* Photosensitized singlet oxygen luminescence from the protein matrix of Zn-substituted myoglobin. *The journal of physical chemistry. A* **118**, 1864-1878, doi:10.1021/jp501615h (2014).

Lindig, B. A. & Rodgers, M. A. J. Rate Parameters for the Quenching of Singlet Oxygen by Water-Soluble and Lipid-Soluble Substrates in Aqueous and Micellar Systems. *Photochemistry and Photobiology* **33**, 627-634, doi:DOI 10.1111/j.1751-1097.1981.tb05468.x (1981).

Michaeli, A. & Feitelson, J. Reactivity of singlet oxygen toward amino acids and peptides. *Photochem Photobiol* **59**, 284-289, doi:10.1111/j.1751-1097.1994.tb05035.x (1994).

Westberg, M., Bregnhoj, M., Etzerodt, M. & Ogilby, P. R. No Photon Wasted: An Efficient and Selective Singlet Oxygen Photosensitizing Protein. *The journal of physical chemistry. B* **121**, 9366-9371, doi:10.1021/acs.jpcb.7b07831 (2017).

Wilkinson, F., Helman, W. P. & Ross, A. B. Rate Constants for the Decay and Reactions of the Lowest Electronically Excited Singlet-State of Molecular-Oxygen in Solution - an Expanded and Revised Compilation. *Journal of Physical and Chemical Reference Data* **24**, 663-1021, doi:Doi 10.1063/1.555965 (1995).


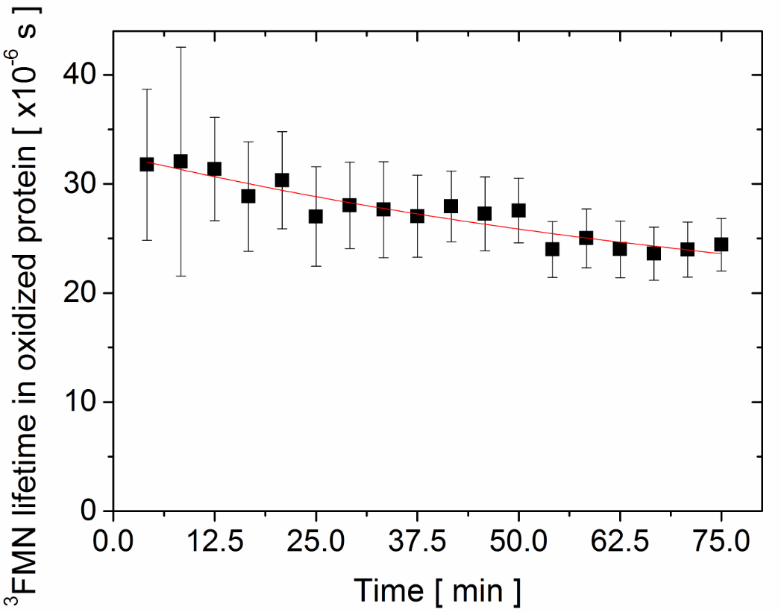
**Figure S1**

**Figure S1.** The values of τ_T_^prot*^ as a function of the irradiation time obtained from fitting transient absorption signals of AsLOV2 C450A. The errors represent 3 times the standard deviation of the fitting parameter.

**Analysis of the model of the irradiation-induced degradation of AsLOV2 C450A**

The set of coupled differential equations describing the concentration of FMN in C450A, C450A* and in the water environment (before bleaching) can be written as follows:

$$\frac{d\left[ \text{FMN} \right]_{\text{prot}}}{dt}=-k_{1}{[\text{FMN}]}_{\text{prot}}$$

$$\frac{d\left[ \text{FMN} \right]_{\text{prot*}}}{dt}=k_{1}{[\text{FMN}]}_{\text{prot}}-k_{\text{2}}{[\text{FMN}]}_{\text{prot*}}$$

$$\frac{d\left[ \text{FMN} \right]_{\text{water}}}{dt}=k_{2}{[\text{FMN}]}_{\text{prot*}}-k_{\text{3}}{[\text{FMN}]}_{\text{water}}$$

The analytical solutions of these equations are:

${[\text{FMN}]}_{\text{prot}}={[\text{FMN}]}_{\text{prot}}^{0}e^{-k_{1}t}$ ….(Eq. S1)

$${[\text{FMN}]}_{\text{prot*}}=\frac{k_{1}{[\text{FMN}]}_{\text{prot}}^{0}}{k_{1}-k_{2}}\left( e^{-k_{2}t}-e^{-k_{1}t} \right)$$

….(Eq. S2)

$${[\text{FMN}]}_{\text{water}}=\frac{k_{1}k_{2}{[\text{FMN}]}_{\text{prot}}^{0}}{k_{1}-k_{2}}\left( \frac{e^{-k_{2}t}-e^{-k_{3}t}}{k_{3}-k_{2}}-\frac{e^{-k_{1}t}-e^{-k_{3}t}}{k_{3}-k_{1}} \right)$$

….(Eq. S3)

**Analysis of the model of the irradiation-induced degradation of AsLOV2 wt**

The observed kinetics of Abs_prot_ and Abs_water_ were analyzed in a semi-empirical way, assuming a linear decrease of the [FMN_prot_] concentration in the studied time range. This assumption is consistent with a constant source term of FMN in water, assigned as K:

$$\frac{d\left[ \text{FMN} \right]_{\text{water}}}{dt}=K-k_{\text{3}}{[\text{FMN}]}_{\text{water}}$$

The solution gives the time-dependence of the FMN concentration in water:

$${[\text{FMN}]}_{\text{water}}=\frac{K}{k_{3}}\left( 1-e^{-k_{3}t} \right)$$

This function was used to fit the Abs_water_ values (black squares) in the Figure 3A, using the same rate of FMN bleaching *k*_3_ as for the mutant (determined in the auxiliary bleaching experiment). It can be seen that the shape of the curve is well described by the bleaching effect.

**References**

Westberg, M., Bregnhoj, M., Etzerodt, M. & Ogilby, P. R. Temperature Sensitive Singlet Oxygen Photosensitization by LOV-Derived Fluorescent Flavoproteins. *The journal of physical chemistry. B* **121**, 2561-2574, doi:10.1021/acs.jpcb.7b00561 (2017).

Torra, J. *et al.* Tailing miniSOG: structural bases of the complex photophysics of a flavin-binding singlet oxygen photosensitizing protein. *Sci Rep* **9**, 2428, doi:10.1038/s41598-019-38955-3 (2019).


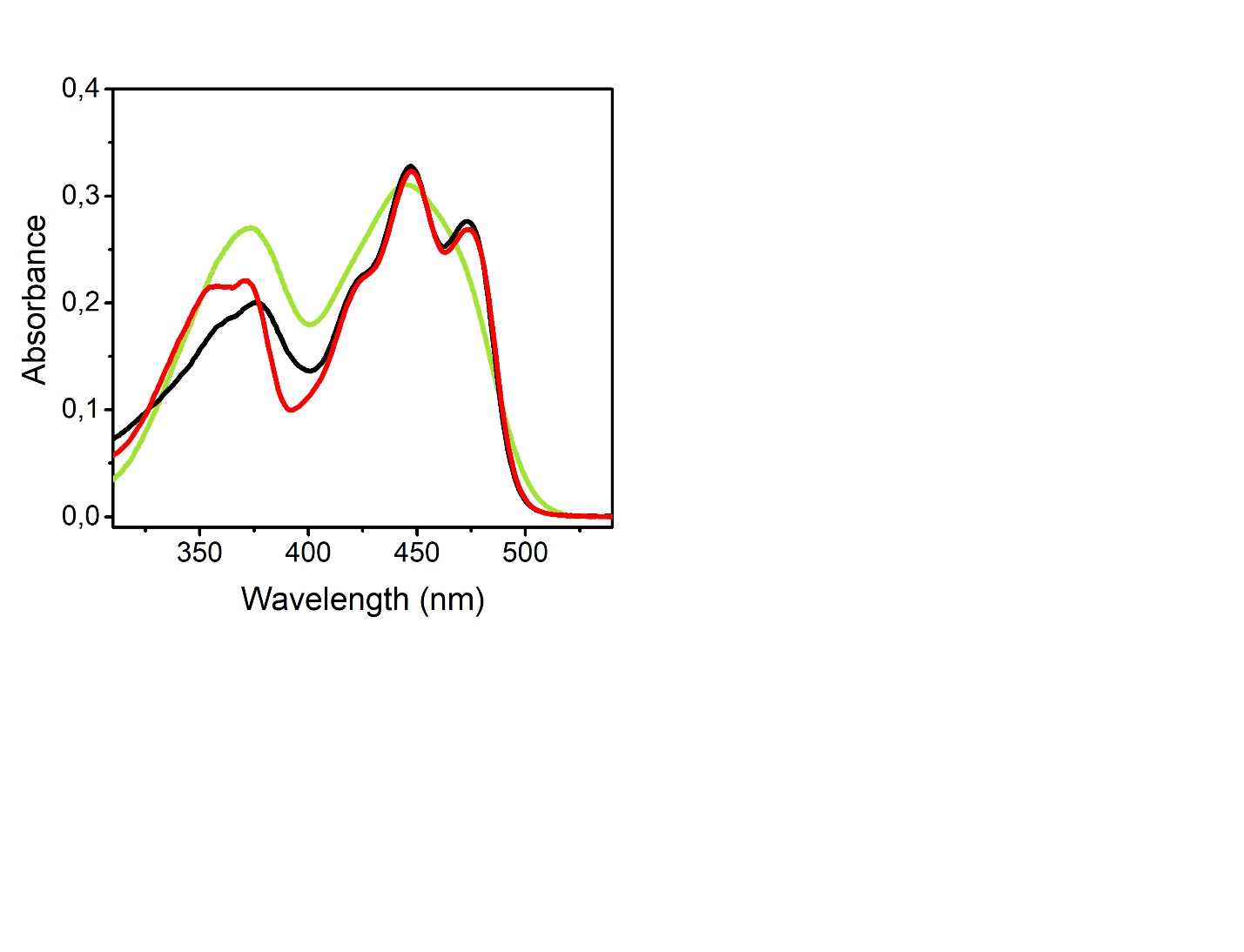
**Figure S2**

**Figure 2S**. Absorption spectra of AsLOV2 wt (black), AsLOV2-C450A (red), and free FMN at 10 μM concentration.

**Figure S2**. Absorption spectra of AsLOV2 wt (**black**), AsLOV2-C450A (**red**), and free FMN (**green**) at 25 μM concentration.


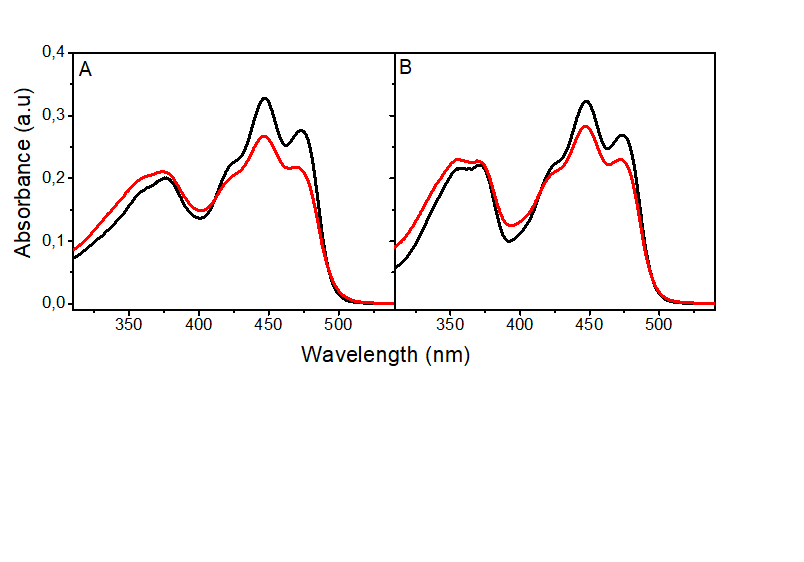
**Figure S3**

**Figure S3**. Absorption spectra of AsLOV2 wt (A) and AsLOV2 C450A (B) before (**black**) and after (**red**) illumination. Proteins concentration was 25 μM.


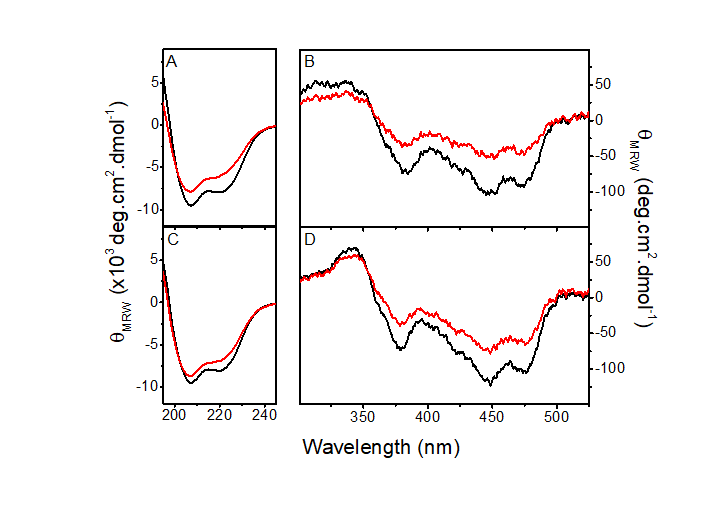
**Figure S4**

**Figure S4**. Circular dichroism spectra of AsLOV2 wt (A, B) and AsLOV2 C450A (C, D) in the far-UV (A, C) and the near-UV and visible regions (B, D) before (**black**) and after (**red**) illumination.

**Figure S5**


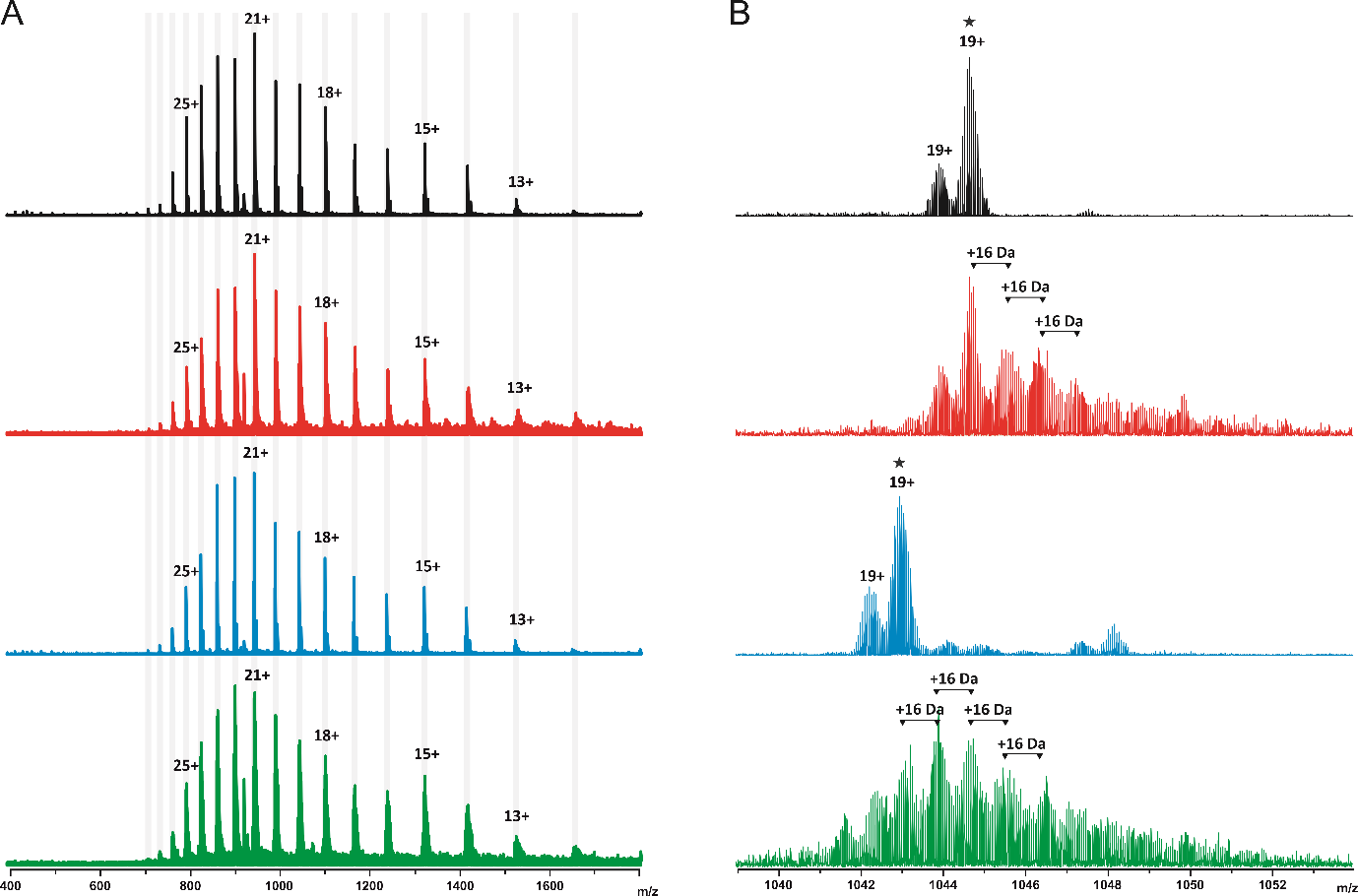


**Figure S5**. Analysis of intact proteins by nESI-FT-ICR MS. **A** – from top to bottom - broad band spectra of wild-type AsLOV2 before (**black**) and after (**red**) irradiation and of C450A form before (**blue**) and after (**green**) irradiation. Clean spectra in panel **A** show classical ESI protein charge state envelope with no signals of peptides/fragments. Panel **B** with zoom on the charge state 19+ demonstrates much higher modification extent in the C450A variant than in the wild-type AsLOV2. Peaks marked with a star symbol are methylated forms of protein.

**Figure S6**


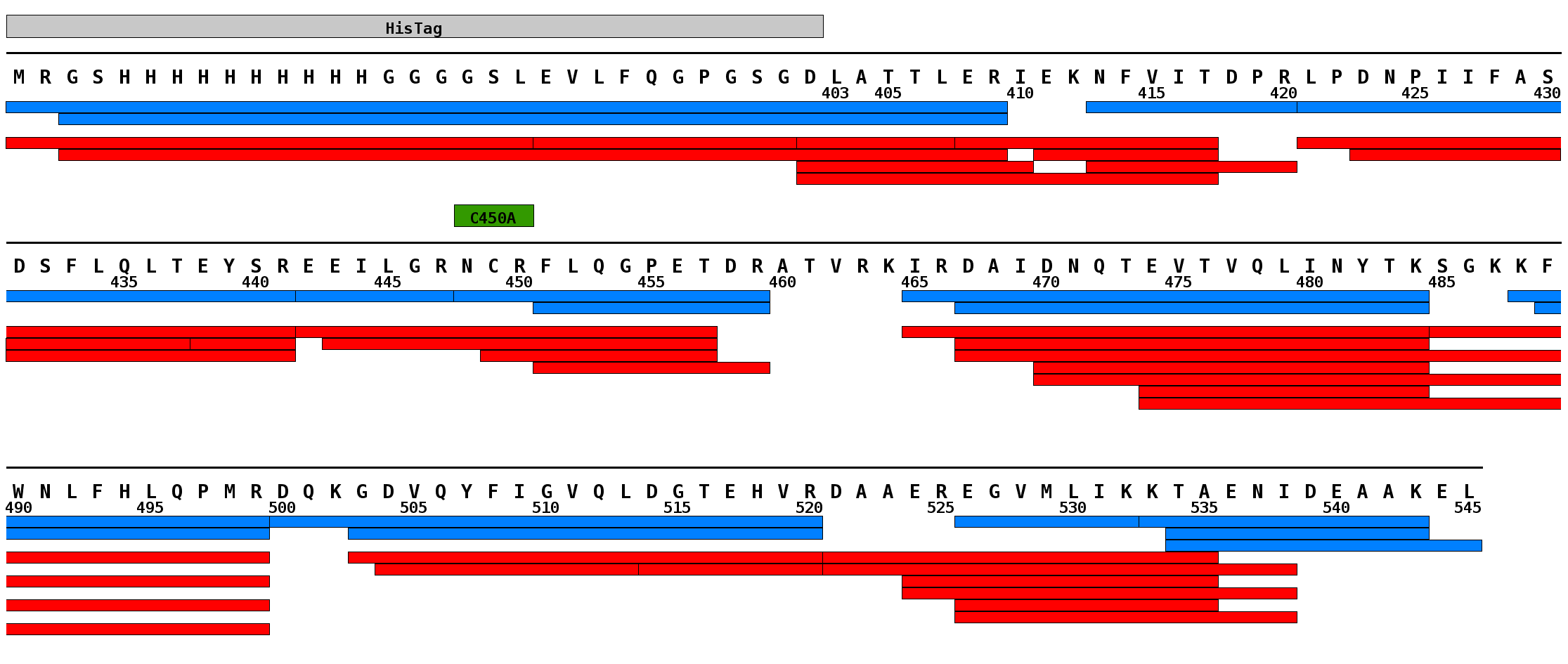


**Figure S6**. Sequence coverage of AsLOV2 obtained in bottom-up experiments. Proteins were digested by trypsin (**blue**) or Asp-N (**red**), analyzed by LC-MS/MS and the data were searched by MASCOT and PEAKS algorithms. Identified peptides are shown as individual bars. The position of **Cys450** is highlighted above the sequence. Sequence coverage for both proteases was 91% and combined coverage is 97%.

**Figure S7**

**Figure S7**. A - AsLOV2 sequence with fragments (b- and y-type ions shown in green and blue, respectively) observed in the top-down MS/MS experiment depicted by black check marks. The site of the C450A mutation is colored in yellow. The His-tag sequence is not numbered and is shown in grey, the native sequence starts with Leu404. Red color highlights residues that were observed in bottom-up as significantly different between the irradiated wt and mut (C450A) form. B - Bar graphs showing quantification of blue-light induced oxidation at selected sites (fragment ions) in both protein forms – wt in red and C450A in green.


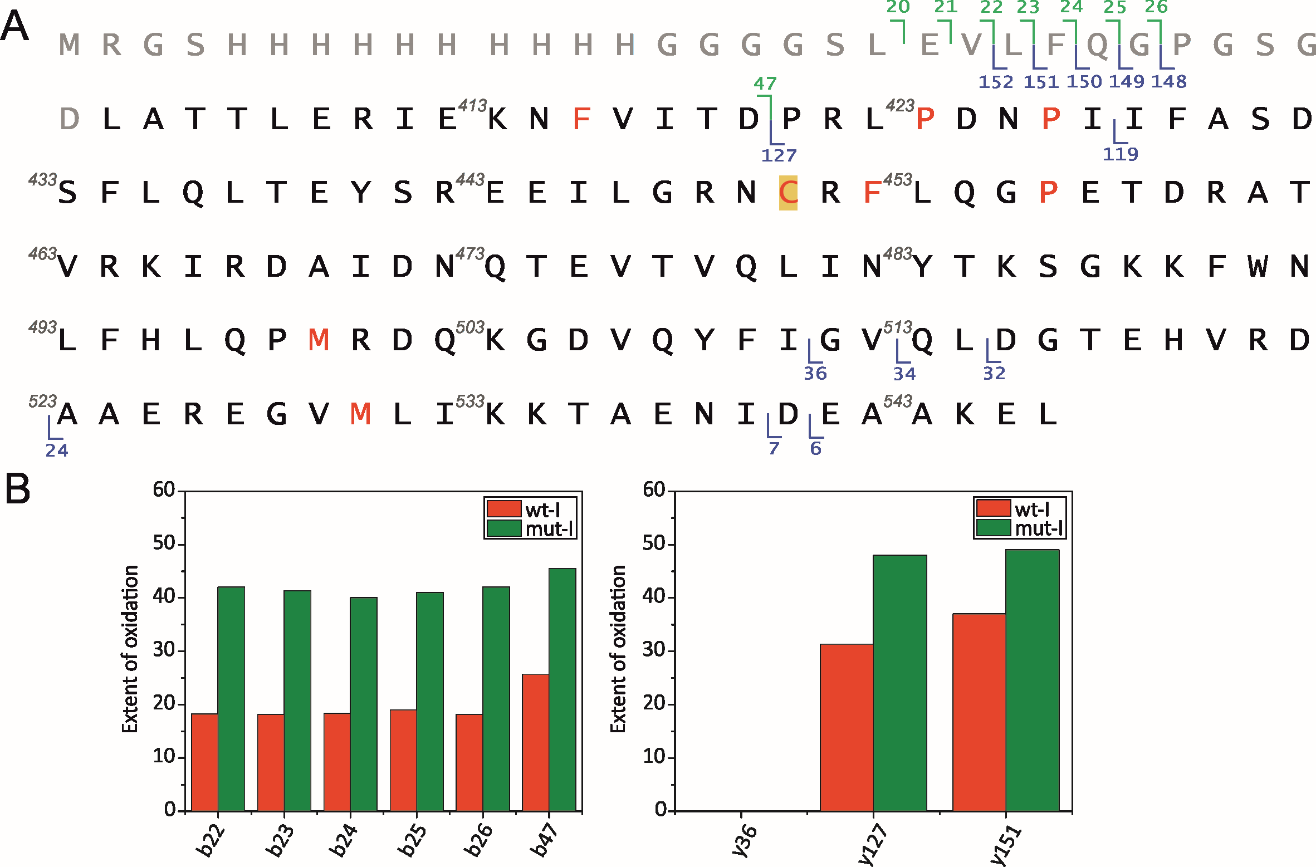


**Figure S8**

**
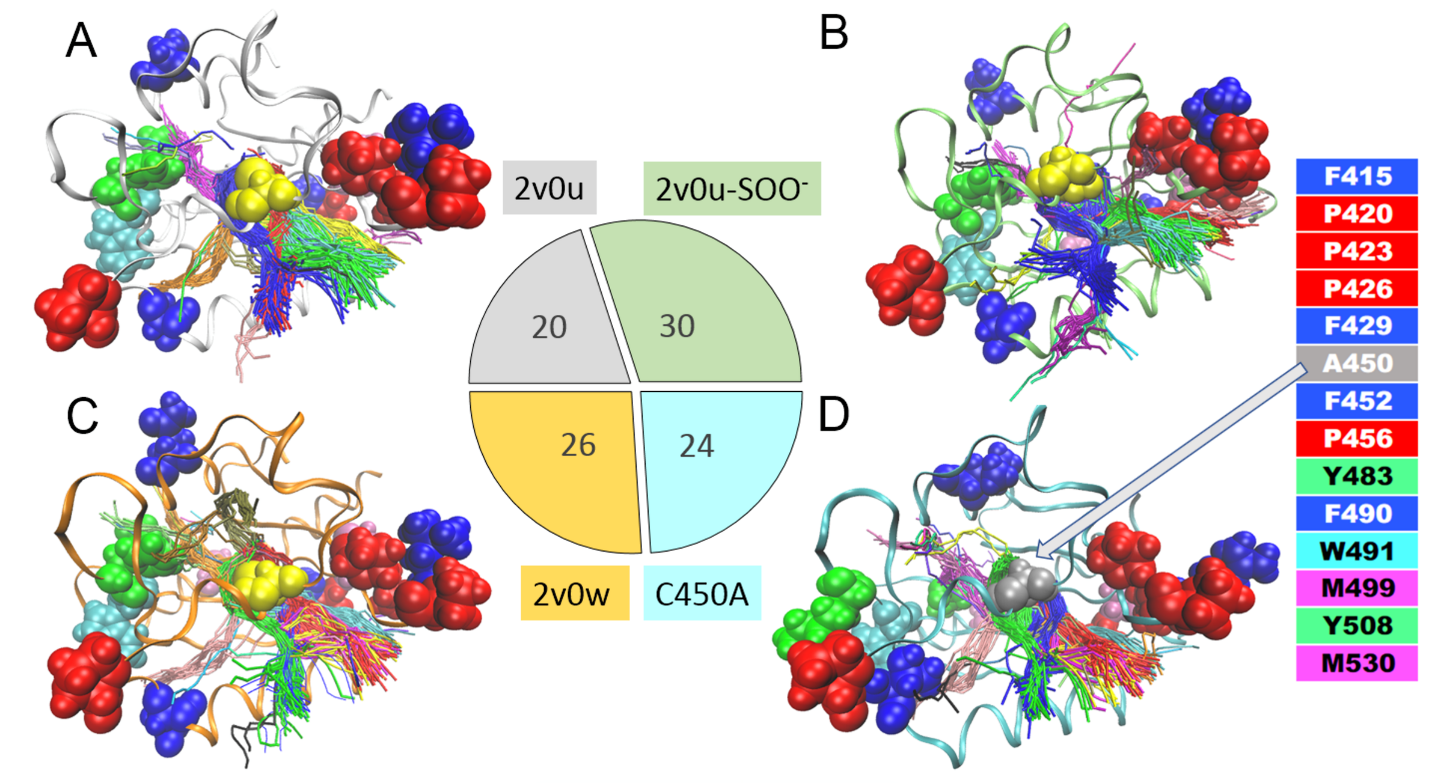
**

**Figure S8**. VMD visualization (<https://www.ks.uiuc.edu/Research/vmd/>) Caver-calculated tunnels resulting from 5ns MD simulations (coordinates saved at 10 ps intervals) of different AsLOV2-related and modified proteins. The color-coded amino acids shown in CPK representation correspond to the amino acids summarized in Figure 6. The C450 atoms are colored yellow; the A450 atoms are in grey. The starting points of all clusters correspond to these amino acids. The color coding of the tunnels represented as sticks do not correspond to the color coding of the amino acids, but are for their distinction only. The backbone of the proteins is shown in ribbon representation. The tunnels were calculated for all 500 saved protein conformations and subsequently clustered with the Caver program. The central pie chart shows the number of clusters for each protein simulated. The tunnels coloring (the tunnels are shown as connected sticks) is arbitrary and do not match the amino acid coloring in the proximity of the tunnels. **A)** simulation based on PDB ID: 2v0u structure. **B)** simulation with *in silico* -SOO^-^ -modified C450 (PDB ID: 2v0u). **C)** PDB ID: 2v0w with chemical bond between C450 and FMN (FMN not shown on the figure). **D)** simulation of 2u0v-C450A mutant of AsLOV2.

**Figure S9**


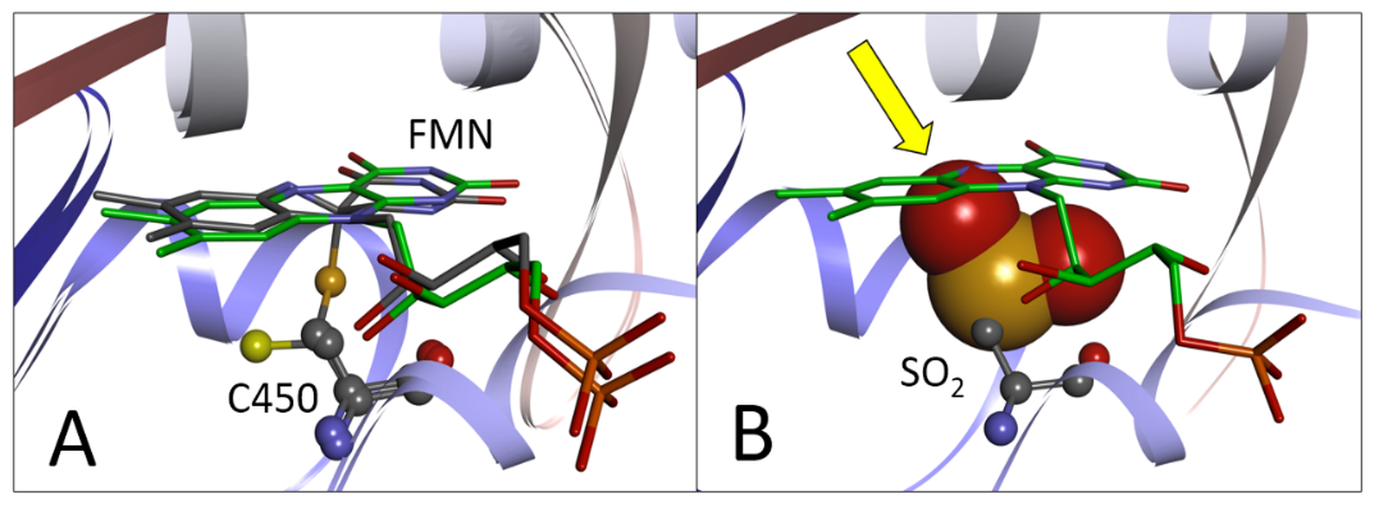


**Figure S9. A)** Superposition of PDB ID: 2w0u (FMN with green carbons) and PDB ID: 2v0w (FMN bound to Cys450 with grey-colored carbons). **B)** -SH to -SO_2_^–^ substitution in C450 with marked atomic clash of the generated structure.


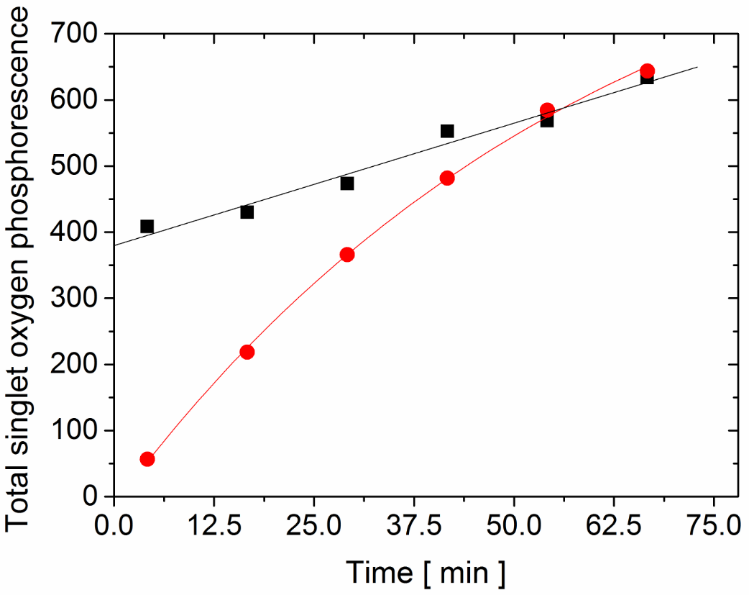
**Figure S10**

**Figure S10**. The total (integrated) ^1^O_2_ phosphorescence signal produced by AsLOV2 C450A (black squares) and AsLOV2 wt (red circles) as a function of the irradiation time. Please note that the production of ^1^O_2_ by AsLOV2 wt practically ends at 25 μs after the laser pulse (Figure 1B), while it lasts well after 40 μs in the case of AsLOV2 C450A (Figure 1A).
